# Metabolomics in early life and the association with body composition at age 2 years

**DOI:** 10.1111/ijpo.12859

**Published:** 2021-10-13

**Authors:** Inge A.L.P. van Beijsterveldt, Stuart G. Snowden, Pernille Neve Myers, Kirsten S. de Fluiter, Bert van de Heijning, Susanne Brix, Ken K. Ong, David B. Dunger, Anita C.S. Hokken‐Koelega, Albert Koulman

**Affiliations:** ^1^ Department of Pediatrics, Subdivision of Endocrinology Erasmus University Medical Center/Sophia Children's Hospital Rotterdam The Netherlands; ^2^ Core Metabolomics and Lipidomics Laboratory, Metabolic Research Laboratories Institute of Metabolic Science, University of Cambridge Cambridge UK; ^3^ Department of Biological Sciences Royal Holloway University of London Egham UK; ^4^ Department of Biotechnology and Biomedicine Technical University of Denmark Kgs. Lyngby Denmark; ^5^ Clinical‐Microbiomics A/S Copenhagen Denmark; ^6^ Danone Nutricia Research Utrecht The Netherlands; ^7^ Medical Research Council Epidemiology Unit University of Cambridge, Institute of Metabolic Science, Cambridge Biomedical Campus Cambridge UK; ^8^ Department of Paediatrics University of Cambridge Cambridge UK

**Keywords:** adiposity, body composition, infants, metabolomics, skinfolds

## Abstract

**Background and Objectives:**

Early life is a critical window for adiposity programming. Metabolic‐profile in early life may reflect this programming and correlate with later life adiposity. We investigated if metabolic‐profile at 3 months of age is predictive for body composition at 2 years and if there are differences between boys and girls and between infant feeding types.

**Methods:**

In 318 healthy term‐born infants, we determined body composition with skinfold measurements and abdominal ultrasound at 3 months and 2 years of age. High‐throughput‐metabolic‐profiling was performed on 3‐month‐blood‐samples. Using random‐forest‐machine‐learning‐models, we studied if the metabolic‐profile at 3 months can predict body composition outcomes at 2 years of age.

**Results:**

Plasma metabolite‐profile at 3 months was found to predict body composition at 2 years, based on truncal: peripheral‐fat‐skinfold‐ratio (T:P‐ratio), with a predictive value of 75.8%, sensitivity of 100% and specificity of 50%. Predictive value was higher in boys (Q^2^ = 0.322) than girls (Q^2^ = 0.117). Of the 15 metabolite variables most strongly associated with T:P‐ratio, 11 were also associated with visceral fat at 2 years of age.

**Conclusion:**

Several plasma metabolites (LysoPC(22:2), dimethylarginine and others) at 3 months associate with body composition outcome at 2 years. These results highlight the importance of the first months of life for adiposity programming.

**Abbreviations**
BCAA: branched chain amino acids
BMI: body mass index
EBF: exclusive breast feeding
EFF: exclusive formula feeding
FDR: false discovery rate
GLM: generalized linear models
HPLC: high performance liquid chromatography
LC–MS: liquid chromatography with mass spectrometry detection
LDL: low‐density lipoprotein
LysoPA: lysophosphatidic acid
LysoPE: lysophosphatidylethanolamine
LysoPG: lysophosphatidylglycerol
LysoPS: lysophosphatidylserine
m/z: mass‐to‐charge ratio
mTOR: mechanistic target of rapamycin
NCD: non‐communicable diseases
PC: phosphatidylcholine
PCA: principal component analysis
PLS‐DA: partial least squares – discriminant analysis
PPAR‐γ: peroxisome proliferator‐activated receptor gamma
S:V‐ratio: subcutaneous:visceral fat ratio
SD: standard deviation
T:P‐ratio: truncal:peripheral skinfold ratio
TLR: toll‐like receptor
UV: unit variance
WHO: World Health Organization

## 1 INTRODUCTION

Childhood obesity is an increasing and worldwide problem. In 1990, 32 million young children had overweight or obesity and this number increased to 41 million in 2016.^1^ Obesity at young age does not only cause short‐term morbidity, but also increases the risk of developing non‐communicable diseases (NCD) in later life, such as insulin resistance, type 2 diabetes and cardiovascular disease.^1^, ^2^

The first months of life are a critical window for metabolic programming affecting adult outcome and body composition.^3^, ^4^ It has been reported that a high weight‐to‐height SD score and a high BMI in childhood are predictive for overweight and obesity in adolescence and adulthood.^5^, ^6^ It is also known however, that a similar body weight or BMI may be accompanied by a different body composition or fat mass percentage, especially in infants and young children.^7^, ^8^ We have previously reported that rapid weight gain in the first 6 months of life is an important risk factor for a higher fat mass in early adulthood^3^ and that infants with a rapid rise in fat mass during the first 6 months of life have higher fat mass percentage trajectories during the first 2 years of life.^9^ Fat mass and its distribution play an important role in the development of unfavourable metabolic outcomes.^10^, ^11^ Especially excessive truncal and visceral fat accumulation compared to peripheral fat is associated with an unfavourable metabolic profile.^12^ The ability to identify infants at risk of obesity at an early stage, will provide the opportunity to develop more targeted preventative strategies.

Also feeding type during the first few months of life influences body composition, with infants receiving exclusive breastfeeding exhibiting different weight trajectories with more subcutaneous fat accumulation and different serum concentrations of appetite regulating hormones compared to infants receiving exclusive formula feeding.^13^, ^14^

An unfavourable body composition with excessive body fat and more visceral fat is associated with an adverse lipoprotein profile in children and adults, especially with high LDL cholesterol levels, which increases the risk of cardiovascular disease.^15^, ^16^ However, not only the standard lipoproteins, but also several hundred lipid species were found in infant plasma.^17^ These could potentially be early biomarkers for unfavourable metabolic outcomes.

Koulman et al. found that the metabolic and lipid profile of exclusively breastfed infants is different from exclusively formula‐fed infants. In breastfed infants, total phosphatidylcholine levels are higher and linoleic acid is less incorporated in palmitate into the phospholipid fraction as compared to that of formula fed infants.^18^, ^19^ In formula‐fed infants, also the amount of formula feeding did influence the metabolic profile. In addition, in infants aged 3 months, phosphatidylcholine (PC) (18:1/16:0) and PC plasmalogen (34:1) were associated with accelerated weight gain, while phosphatidylcholine (20:4/18:0), PC plasmalogen (36:4), Sphingomyelin (d18:1/16:0) had an association with poor weight gain.^18^, ^20^

These differences in metabolic profile and phospholipid composition could change the endogenous lipid metabolism and, thus, have consequences for adiposity programming and vice versa. We, therefore, hypothesized that specific metabolites in early life associate with body composition parameters at 2 years. The primary objective was to investigate if metabolites at 3 months of age are associated with, and even may predict specific body composition outcomes at age 2 years in a cohort of healthy infants. Second, we aimed to investigate if any metabolites, predictive of 2 years body composition, already associated with body composition parameters at 3 months. Lastly, we investigated if the metabolite profile at 3 months was different between boys and girls and between infants with exclusive breastfeeding and those with exclusive formula feeding.

## 2 MATERIAL AND METHODS

### 2.1 Subjects

The cohort consisted of infants participating in the Sophia Pluto study, an ongoing birth cohort study of healthy infants, aimed to provide detailed data on early growth‐ and body composition trajectories in infancy and childhood. Infants were recruited between January 2013 and November 2017, from several maternity wards in Rotterdam, the second largest city in the Netherlands. All participants met the following inclusion criteria: born term (≥37 weeks of gestation), an uncomplicated neonatal period, without severe asphyxia (defined as an Apgar‐score below 3 after 5 min), sepsis or the need for respiratory ventilation.

Exclusion criteria were maternal disease or medication that could interfere with fetal growth, including maternal corticosteroids, insulin‐dependent (gestational) diabetes mellitus, known congenital or postnatal disease or intrauterine infection that could interfere with infant growth. The Medical Ethics Committee of Erasmus Medical Centre approved the study. We obtained written informed consent of all parents/caregivers with parental authority.

### 2.2 Data collection and measurements

Trained staff carried out the measurements according to standard procedures at 3 months and at 2 years. Birth data were taken from medical records. Parental characteristics and feeding type were obtained by standardized interviews at the clinic visits and by questionnaires. Details about frequency and amount of infant feeding and dates of changes in feeding mode were recorded. Exclusive breastfeeding (EBF) was defined as receiving only mother's milk until at least the age of 3 months. Exclusive formula feeding (EFF) was defined as receiving only infant formula starting before the age of 1 month. Mixed feeding (mix) was defined as starting with formula feeding between 1 and 3 months of age.

### 2.3 Anthropometrics

Weight was measured to the nearest 5 g by an electronic infant scale (Seca 717, Hamburg, Germany). Length was measured twice in supine position to the nearest 0.1 cm by an infantometer (Seca 416). BMI was calculated as weight (kg)/length^2^ (m^2^). Head, waist and hip circumference were measured to the nearest 0.1 cm by a circumference measuring tape (Seca 201). Skinfolds were measured to the nearest mm with a skinfold calliper (Slimguide C‐120, Creative Health) at every visit on four sites on the left side of the body: biceps, triceps, subscapular and suprailliac. The intra‐observer intra class correlation coefficient (ICC) and inter‐observer ICC were determined earlier; 0.88 and 0.76, resp..^21^ Peripheral skinfolds were calculated as triceps + biceps. Truncal skinfolds were calculated as subscapular + suprailliac.^21^ The truncal:peripheral skinfold ratio (T:P‐ratio) was calculated as truncal skinfolds divided by peripheral skinfolds.

SD‐scores of weight, length and weight‐for‐length were calculated using Growth Analyser software (http://www.growthanalyser.org).^22^

### 2.4 Abdominal fat

Abdominal visceral and subcutaneous fat were determined at 3 months and 2 years, using ultrasound (Prosound 2 ultrasound with a UST‐9137 convex transducer [both Hitachi Aloka Medical, Zug, Switzerland]). Fat depths were measured in supine position, with the transducer on the intercept of the xiphoid line and the waist circumference measurement plane. Visceral fat was measured in the longitudinal plan from the peritoneal boundary to the corpus of the lumbar vertebra with a probe depth of 9 cm and abdominal subcutaneous fat in the transvers plan from the cutaneous boundary to the linea alba with a probe depth of 4 cm. Minimal pressure was applied. Validity and reproducibility of measurements were confirmed in the Cambridge Baby Growth Study (CBGS), the relative interobserver technical error of measurement was 3.2% for visceral fat and 3.6% for subcutaneous fat.^23^ If the vertebra were not visualized, measurements were considered unsuccessful and were excluded from analyses. The abdominal subcutaneous:visceral fat ratio (S:V‐ratio) was calculated as abdominal subcutaneous fat divided by visceral fat.

### 2.5 Sample collection

Blood samples were collected at 3 months and 2 years by capillary toe or finger prick sampling after the infants had fasted for a minimum of 2 h. Blood was collected in heparin microtubes (BD Microtainer®, 200–400 μl) and centrifuged to prepare plasma. The samples were stored at –80°C until analysis. Plasma samples were transported on dry ice to the University of Cambridge (UK) for metabolic profiling.

### 2.6 Metabolic profiling

Metabolic profiling was performed with high throughput platform in the Metabolic Research Laboratories of the Institute of Metabolic Science in Cambridge. The samples were analysed using liquid chromatography mass spectrometry method^24^ ultimately yielding results of the absolute and relative concentration of 349 individual metabolites and lipids. The protein precipitation liquid extraction protocol was used as described previously.^24^ Briefly, 50 μl of plasma was transferred into a 2 ml screw cap Eppendorf plastic tube (Eppendorf, Stevenage, UK). Immediately, 650 μl of chloroform was added to each sample, followed by thorough mixing. Then, 100 μl of the internal standards (5 μM in methanol), 100 μl of the carnitine internal standards (5 μM in methanol) and 150 μl of methanol was added to each sample, followed by thorough mixing, after which 400 μl of acetone was added to each sample. The samples were vortexed and centrifuged for 10 min at ~20 000 g to pellet any insoluble material. The supernatant was pipetted into separate 2 ml screw cap amber‐glass auto‐sampler vials (Agilent Technologies, Cheadle, UK). The organic extracts were evaporated to dryness using a Concentrator Plus system (Eppendorf, Stevenage, UK) run for 60 min at 60°C. The samples were reconstituted in 100 μl of a 2:1:1 mixture of propan‐2‐ol, acetonitrile and water, and then thoroughly vortexed. The reconstituted sample was transferred into a 250 μl low‐volume vial insert inside a 2 ml amber glass auto‐sample vial ready for liquid chromatography with mass spectrometry detection (LC–MS) analysis.

Chromatographic separation was achieved using Shimadzu HPLC System (Shimadzu UK Limited, Milton Keynes, UK) with the injection of 10 μl onto a Waters Acquity UPLC® CSH C18 column (Waters, Hertfordshire, UK); 1.7 μm, I.D. 2.1 × 50 mm^2^, maintained at 55°C. Mobile phase A was 6:4 acetonitrile and a 10 mM ammonium formate solution in water. Mobile phase B was 9:1 propan‐2‐ol and a 10 mM ammonium formate solution in acetonitrile. The flow was maintained at 500 μl/min through the following gradient: 0.00 min_40% mobile phase B; 0.40 min_43% mobile phase B; 0.45 min_50% mobile phase B; 2.40 min_54% mobile phase B; 2.45 min_70% mobile phase B; 7.00 min_99% mobile phase B; 8.00 min_99% mobile phase B; 8.3 min_40% mobile phase B; and 10 min_40% mobile phase B. The sample injection needle was washed using 9:1, propan‐2‐ol and acetonitrile. The mass spectrometer used was the Thermo Scientific Exactive Orbitrap with a heated electrospray ionization source (Thermo Fisher Scientific, Hemel Hempstead, UK). The mass spectrometer was calibrated immediately before sample analysis using positive and negative ionization calibration solution (recommended by instrument manufacturer). Additionally, the mass spectrometer scan rate was set at 4 Hz, giving a resolution of 25 000 (at 200 m/z) with a full‐scan range of m/z 100–1800 with continuous switching between positive and negative mode.

### 2.7 Data processing

All .RAW files were converted to .mzXML files using msConvert (ProteoWizard).^25^ Converted files were subsequently processed in R (v3.3.1) using the CAMERA package^26^ with peak picking performed using the centWave method as this enables for the deconvolution of closely eluting and slightly overlapping peaks. Metabolite variables included within the final dataset were defined as peaks that had an intensity at least three times higher in analytical samples relative to the extraction blanks and that was present in at least 90% of the analysed samples. If possible, metabolite variables were putatively annotated by matching measured accurate masses to entities in the Human Metabolome database (www.hmdb.ca).

### 2.8 Statistical analysis

Clinical characteristics are expressed as mean and standard deviation (SD) or as median and interquartile range (IQR) when not normally distributed. Differences in clinical characteristics were determined by independent Student's *t* test or Mann–Whitney *U*‐test for non‐parametric parameters. Pearson's correlation coefficient was used to determine bivariate correlations. Exact power calculations for this type of experiments were not readily available at the design of the project. Previous analyses of the lipid profiles in the Cambridge Baby Growth Study showed significant associations with catch‐up growth^18^ using around 215 samples. By using the Sophia Pluto cohort, that is almost double in sample size, sufficient power was considered to find metabolites that are associated with fat distribution.

Using WHO classification, overweight was defined as a weight‐for‐length > 2 SDS and obesity was defined as a weight‐for‐length > 3 SDS.^1^ Underweight was defined as an weight‐for‐age < −2 SDS.^27^

To analyse the association between metabolite profile and six measures of body composition, peripheral and truncal fat, subcutaneous and visceral fat and the ratio of truncal:peripheral fat and the ratio of abdominal subcutaneous:visceral fat, individuals were stratified into tertiles of each body composition measure (‘high’, ‘middle’ and ‘low’). Multivariate analysis was performed using principal component analysis (PCA). Partial least squares – discriminant analyses (PLS‐DA), performed in SIMCA v13.0 (Umetrics, Umeå, Sweden), were used to identify associations between the metabolite profiles generated from samples collected at 3 months of age and body composition at 2 years of age, with all data logarithmically transformed (base10) and scaled to unit variance (UV) in all models. The performance of the generated models was based on cumulative correlation coefficients R^2^X[cum] and R^2^Y[cum] (PLS‐DA only) to assess what percentage of the variation in the X and Y variables was explained by the model. The predictive performance of these models was based on the 7‐fold cross validation Q^2^[cum] and the significance of the model was determined using ANOVA of the cross validation residuals (CV‐ANOVA).

To estimate if it is possible to determine body composition at 2 years of age using metabolite profile data from 3 months of age, random forest machine learning models were performed in R (V3.3.1). Each of the body compositions measures were split into a training (70%) and testing set (30%). The performance of these models was assessed by looking at overall classification accuracy of predicating ‘high’ or ‘low’ body composition measures, as well as the sensitivity and specificity of the predictions made in the testing set. Univariate analysis of metabolites of interest was performed using generalized linear models (GLM) calculated in R (v3.3.1). We corrected for possible confounders: sex, birth weight and feeding type. Additional corrections for BMI SDS at age 3 months, weight‐for‐length SDS and total skinfolds at age 2 years did not change the results. To determine differences between boys and girls and between the different types of feeding type, models were performed for boys and girls separately and for EBF, EFF and mixed feeding separately.

Controlling for the false discovery rate (FDR) was done by calculating a Bonferroni corrected *p*‐threshold based on all 600 metabolite variables (*p* = 8.33 × 10^−5^).

## 3 RESULTS

The study group consisted of 318 healthy infants of the Sophia Pluto cohort with complete body composition data and blood samples. One hundred forty‐three (45%) were girls and 66.4% of the infants was Caucasian. Clinical characteristics are presented in Table 1. Of all infants, 38.7% received exclusive breastfeeding (EBF) until the age of 3 months and 25.5% of the infants were exclusive formula fed (EFF). This did not differ between boys and girls. Body composition parameters were not different between boys and girls, except for visceral fat at 3 months, which is higher in boys than in girls (*p* = 0.017). Abdominal subcutaneous and visceral fat and truncal:peripheral fat ratio (T:P‐ratio) ratio decreased over time from age 3 months to 2 years. Based on the WHO criteria for weight‐for‐length SDS, 93.1% of the infants had normal weight, 5.7% was underweight and 1.3% had overweight at 2 years of age. None of the infants classified had obesity. This was not different between boys and girls. Infants were divided in tertiles based on T:P‐ratio (Table 2).

**TABLE 1:** Clinical characteristics

	All *N* = 318	Boys *N* = 175	Girls *N* = 143	*p*‐value
Gestational age (weeks)	39.74 (1.21)	39.66 (1.28)	39.83 (1.13)	0.224
Sex (%)		55.0	45.0	0.479
Birth weight SDS	0.28 (1.15)	0.42 (1.08)	0.11 (1.20)	**0.017**
Birth length SDS^a^	0.68 (1.20)	0.83 (1.19)	0.49 (1.19)	0.058
Ethnicity (%)				0.060
Caucasian	211 (66.4%)	122 (69.7%)	89 (62.2%)	
Black	21 (6.6%)	5 (2.9%)	16 (11.2%)	
Asian	3 (0.9%)	1 (0.6%)	2 (1.4%)	
Latin	1 (0.3%)	1 (0.6%)	0	
Other	64 (20.1%)	35 (20.0%)	29 (20.3%)	
Missing	18 (5.7%)	11 (6.2%)	7 (4.9%)	
Mode of delivery				0.479
Vaginal	219 (68.9%)	115	94	
Caesarean Section	98 (30.8%)	59	39	
Missing	1 (0.3%)	1	0	

3 months	*N* = 318	*N* = 175	*N* = 143	
Age (months)	2.99 (2.92–3.09)	2.99 (2.92–3.09)	2.99 (2.92–3.06)	0.615
Feeding mode				0.597
Exclusive breastfeeding	123 (38.7%)	64	59	
Exclusive formula feeding	81 (25.5%)	48	33	
Mix feeding	113 (35.5%)	62	51	
Weight‐for‐length SDS	0.22 (1.01)	0.27 (0.95)	0.15 (1.08)	0.306
Length SDS	0.35 (0.87)	0.49 (0.81)	0.17 (0.92)	**0.001**
Peripheral skinfolds (mm)	15 (13–16)	15 (13–17)	14 (13–16)	0.139
Truncal skinfolds (mm)	13 (11.5–16)	13 (11–16)	14 (12–17)	0.633
T:P‐ratio	0.93 (0.81–1.09)	0.92 (0.81–1.07)	1.00 (0.81–1.11)	0.112
Abdominal subcutaneous fat (cm)	0.41 (0.32–0.49)	0.41 (0.34–0.49)	0.40 (0.32–0.49)	0.787
Visceral fat (cm)	2.36 (0.57)	2.42 (0.58)	2.29 (0.54)	**0.048**
S:V‐ratio	0.17 (0.13–0.22)	0.16(0.13–0.21)	0.18(0.14–0.23)	0.111

2 years	*N* = 318	*N* = 175	*N* = 143	
Age (months)	24.02 (23.92–24.25)	24.02 (23.92–24.21)	24.08 (23.92–24.35)	0.119
Weight‐for‐length SDS	−0.41 (1.06)	−0.41 (1.13)	−0.41 (0.97)	0.945
Underweight	18 (5.7%)	10	8	0.720
Normal	296 (93.1%)	162	134	
Overweight	4 (1.3%)	3	1	
Obese	0	0	0	
Length SDS	0.22 (1.01)	0.28 (1.04)	0.15 (0.96)	0.250
Peripheral skinfolds (mm)	16 (14–19)	16 (14–19)	16 (14–18)	0.741
Truncal skinfolds (mm)	13 (11–15)	13 (11–15)	13 (11–15)	0.994
T:P‐ratio	0.79 (0.67–0.93)	0.79 (0.68–0.94)	0.78 (0.65–0.92)	0.686
Abdominal subcutaneous fat (cm)	0.32 (0.26–0.40)	0.32 (0.26–0.40)	0.34 (0.26–0.39)	0.787
Visceral fat (cm)	2.12 (1.82–2.51)	2.11 (1.77–2.49)	2.14 (1.84–2.56)	0.387
S:V‐ratio	0.16 (0.13–0.22)	0.16 (0.12–0.21)	0.15 (0.12–0.20)	0.280

*Note*: Data expressed as mean (SD) or median (IQR). Significant *p*‐values are boldfaced.

Abbreviations: BMI, body mass index; S:V‐ratio = subcutaneous:visceral fat ratio; SDS, standard deviation score; T:P‐ratio, Truncal: peripheral skinfold ratio.

^a^ Birth weight SDS *n* = 175.

**TABLE 2:** Clinical characteristics and body composition of infants with high, middle and low trunk: peripheral fat ratio

	Low	Middle	High
T:P‐ratio (range)	<0.72	0.72–0.88	>0.88
T:P‐ratio (mean ± SD)	0.62 ± 0.07	0.79 ± 0.05	1.03 ± 0.12
Sex (F/M)	51/55	39/49	43/60
Feeding type (EBF/EFF/Mix)	39/30/37	36/23/29	39/25/39
3 months			
Peripheral skinfolds (mm)	14.48 ± 2.54	14.95 ± 2.78	14.96 ± 2.94
Trunk skinfolds (mm)	12.97 ± 3.04^a^	14.47 ± 3.15^a^	14.76 ± 3.87^a^
Total skinfolds (mm)	27.45 ± 5.21^a^	29.42 ± 5.70^a^	29.72 ± 9.93^a^
Abdominal subcutaneous fat (cm)	0.41 ± 0.11	0.40 ± 0.11	0.43 ± 0.12
Visceral fat (cm)	2.41 ± 0.56	2.35 ± 0.57	2.34 ± 0.57
2 years			
Peripheral skinfolds (mm)	18.13 ± 6.25^a^	16.98 ± 5.23^a^	14.77 ± 4.77^a^
Trunk skinfolds (mm)	11.18 ± 6.22^a^	13.36 ± 6.85^a^	15.12 ± 6.69^a^
Total skinfolds (mm)	29.31 ± 5.25	30.34 ± 5.23	29.89 ± 4.77
Abdominal subcutaneous fat (cm)	0.32 ± 0.09	0.33 ± 0.09	0.36 ± 0.10
Visceral fat (cm)	2.24 ± 0.64	2.27 ± 0.57	2.08 ± 0.51^a^

Abbreviations: EBF, exclusively breastfed; EFF, exclusively formula fed; F, female; M, male; Mix, mixed fed; SD, standard deviation; T:P‐ratio, truncal: peripheral skinfold ratio.

^a^ Indicates a significant (*p* < 0.05) difference between groups. All *p*‐values were calculated using generalized linear models applied simultaneously to all three groups.

### 3.1 Association between metabolite variables at 3 months and body composition at 2 years

**FIGURE 1:**
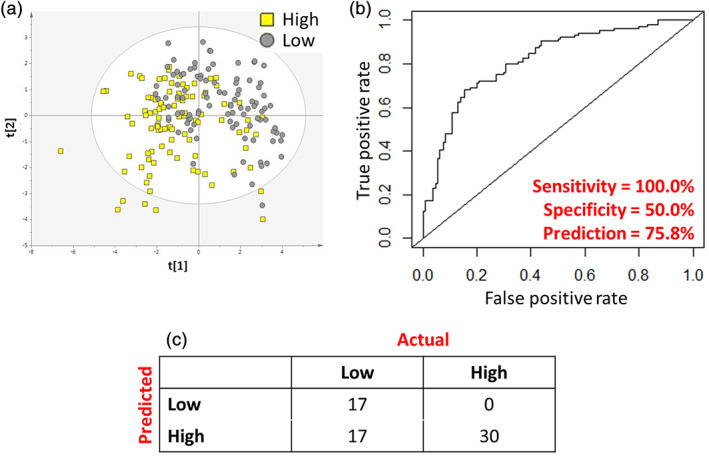
Evaluation of the ability for 3 month metabolite profile to predict truncal:peripheral fat ratio at 2 years. (A) Scores plot of PLS‐DA model calculated on individuals with ‘high’ and ‘low’ truncal:peripheral fat ratio after the dataset had been stratified into three groups of ‘high’, ‘middle’ and ‘low’ truncal:peripheral fat ratio. (B) Receiver operating characteristic curve showing diagnostic ability of the model to identify individuals with high and low truncal: peripheral fat ratio. (C) Prediction matrix showing rates of correct classification

There was a modest association between the plasma metabolite profile at 3 months of age and body composition at 2 years of age, measured as truncal:peripheral ratio (T:P‐ratio) (R^2^X = 0.224, R^2^Y = 0.351, Q^2^ = 0.185, CV‐ANOVA = 5.71х10^−8^) (Figure 1). Using random forest, modest predictions for infants with high and low T:P‐ratio at 2 years were achieved using 3 month plasma metabolite profiles with an predictive performance of 75.8%, a sensitivity of 100% and a specificity of 50.0%. Meaning that 100% of the infants with a high T:P‐ratio at 2 years of age was predicted to have a high T:P‐ratio based on their metabolite profile at 3 months, while 50.0% of the infants with a low T:P‐ratio prediction based on their plasma metabolite profile did have a low T:P‐ratio measured at 2 years of age.

Of the 15 most strongly associated metabolite variables with T:P‐ratio at 2 years of age, two passed ‘false discover rate’ (FDR) correction based on a Bonferroni corrected *p*‐threshold (*p* = 8.33 × 10^−5^) and eight passed Benjamini‐Hochberg based on all 600 variables (Table 3). Of these, nine metabolites were annotated: Lysophosphatidylserine 22:2 (LysoPS (22:2)) had a fold change of 1.48 (*p* = 2.32 × 10^−5^) in infants with a high T:P‐ratio compared to infants with low T:P‐ratio. Meaning that the relative abundance of LysoPS (22:2) was 48% higher in infants with a high T:P‐ratio at 2 years, compared to infants with low a T:P‐ratio at 2 years of age. For dimethylarginine, esterone glucoronide (C_24_H_30_O_8_)_,_ the C13 isotope of hydroxypentaoxolanostenoic acid (C_30_H_40_O_8_), hydroxyprogesterone glucoronide (C_27_H_38_O_9_), lysophosphatidylethanolamine (20:1) (LysoPE(20:1)) the fold changes were 1.85 (*p* = 0.0002), 1.65 (*p* = 0.0003), 1.31 (*p* = 0.0003), 1.31 (*p* = 0.0007) and 1.09 (*p* = 0.0005), resp. Other annotated metabolites were lysophosphatidylglycerol (16:0) (LysoPG(16:0)), C_30_H_40_O_8_ and lysophosphosphatidic acid (22:1) (LysoPA (22:1)). All had a fold change above 1.

**TABLE 3:** Panel of 15 plasma metabolite variables at 3 months most strongly associated with truncal: peripheral fat ratio at 2 years

			All Individuals			Boys		Girls	
Metabolite variables	m/z	Retention time (min)	*p*‐value	Corrected *p*‐value	Fold change	*p*‐value	Fold change	*p*‐value	Fold change
LysoPS(22:2)	578.3481	0.49	5.99 × 10^−06^ ^a^ ^,^ ^b^	2.16 × 10^−05^ ^a^ ^,^ ^b^	1.48	4.95 × 10^−05^	1.58	0.021	1.37
Unkown	1015.6761	0.39^a^ ^,^ ^b^	2.28 × 10^−05^ ^a^ ^,^ ^b^	3.79 × 10^−05^ ^a^ ^,^ ^b^	1.57	0.0002	1.64	0.033	1.49
Dimethylarginine	203.0533	0.33	0.0001^b^	0.0002^b^	1.85	0.0001	2.20	0.074	1.50
C_24_H_30_O_8_	447.2815	0.47	0.0002^b^	0.0003^b^	1.65	0.0009	1.80	0.032	1.49
C^13^ isotope of C_30_H_40_O_8_	530.3357	0.52	0.0003^b^	0.0003^b^	1.31	0.0069	1.26	0.016	1.36
C_27_H_38_O_9_	507.3104	0.50	0.0004^b^	0.0007^b^	1.31	0.0015	1.36	0.072	1.26
Unknown	431.2617	0.39	0.0005^b^	0.0007^b^	1.38	0.0003	1.50	0.104	1.26
LysoPE(20:1)	508.3468	0.66	0.0008^b^	0.0005^b^	1.09	0.0053	1.08	0.059	1.09
Unknown	590.3393	0.47	0.0018	0.0017	1.48	0.0063	1.49	0.052	1.47
LysoPG(16:0)	486.2760	0.54	0.0022	0.0026	1.14	0.0240	1.11	0.054	1.17
C_30_H_40_O_8_	529.3246	0.47	0.0034	0.0034	1.60	0.0075	1.73	0.114	1.46
LysoPA(22:1)	495.3132	0.86	0.0040	0.0051	1.41	0.0122	1.42	0.101	1.39
Unknown	200.8896	0.319	0.0043	0.0031	0.91	0.0066	0.91	0.069	0.90
Unknown	162.9649	0.367	0.0046	0.0075	2.03	0.0021	2.76	0.418	1.29
Unknown	222.8750	0.38	0.0050	0.0022	0.87	0.1124	0.95	0.006	0.79

*Note*: ‘*p*‐value’ are the unadjusted *p*‐values. ‘Corrected *p*‐values’ have been adjusted for sex, birth weight and feeding type. All *p*‐values were calculated using generalized linear models comparing high and low tertiles of trunk: peripheral ratio. Fold change is calculated relative to the ‘low’ group. Putative annotations: C_24_H_30_O_8_ – Esterone Glucoronide, C_27_H_38_O_9_ –Hydroxyprogesterone Glucoronide, C_30_H_40_O_8_ – Hydroxypentaoxolanostenoic acid.

Abbreviations: LysoPA, lysophosphosphatidic acid; LysoPE, lysophosphatidylethanolamine; LysoPG, lysophosphatidylglycerol; LysoPS, lysophosphatidylserine; m/z, mass‐to‐charge ratio.

^a^ Passing FDR based on Bonferoni.

^b^ Passing FDR based on Benjamini‐Hochberg.

Having identified candidate biomarkers at age 3 months for the T:P‐ratio at 2 years of age, we wanted to determine the inter‐relation between the biomarkers, body composition and potentially confounding factors. After correcting for sex, birth weight and feeding type (Table 3) all biomarkers remained significant (*p* < 0.05) with two passing a Bonferroni corrected *p*‐threshold and six passing Benjamini–Hochberg correction. Additional corrections for BMI SDS at age 3 months, weight‐for‐length SDS and total skinfolds at age 2 years did not change the results (data not shown). Of the 15 metabolite variables, four were already associated with T:P ratio at 3 months of age (*p* < 0.05) (Table S1).

Eleven of the 15 metabolite variables, identified at age 3 months, were significantly associated with visceral fat at 2 years of age (*p* < 0.05), though none passed FDR based on either Bonferroni or Benjamini‐Hochberg (Table 4). All had a fold change below 1, meaning that the relative abundance of these metabolite variables was lower at 3 months in infants with higher visceral fat at 2 years. T:P‐ratio and visceral fat were not correlated, R = −0.073 (*p* = 0.220). We also found that some of the 15 metabolite variables were associated with peripheral and trunk fat levels although none of these associations passed FDR (data not shown).

**TABLE 4:** Panel of 15 plasma metabolite variables at 3 months associated with visceral fat at 2 years

	All Individuals			Boys		Girls	
	*p*‐value	Corrected *p*‐value	Fold change	*p*‐value	Fold change	*p*‐value	Fold change
LysoPS (22:2)	0.003	0.004	0.75	0.084	0.80	0.010	0.68
Unkown	0.007	0.008	0.72	0.109	0.78	0.026	0.64
Dimethylarginine	0.002	0.003	0.62	0.032	0.62	0.025	0.61
C_24_H_30_O_8_	0.009	0.010	0.71	0.105	0.74	0.028	0.66
C^13^ isotope of C_30_H_40_O_8_	0.005	0.008	0.79	0.108	0.83	0.015	0.73
C_27_H_38_O_9_	0.014	0.016	0.81	0.191	0.86	0.025	0.74
Unknown	0.209	0.207	0.88	0.209	0.85	0.523	0.91
LysoPE (20:1)	0.081	0.096	0.95	0.124	0.95	0.299	0.95
Unknown	0.023	0.028	0.74	0.142	0.77	0.071	0.69
LysoPG (16:0)	0.046	0.036	0.89	0.332	0.93	0.077	0.83
C_30_H_40_O_8_	0.002	0.002	0.60	0.046	0.64	0.010	0.55
LysoPA(22:1)	0.042	0.051	0.77	0.236	0.82	0.079	0.71
Unknown	0.435	0.459	1.03	0.518	1.02	0.595	1.04
Unknown	0.001	0.003	0.49	0.007	0.44	0.090	0.55
Unknown	0.715	0.627	0.98	0.203	0.93	0.511	1.06

*Note*: ‘*p*‐value’ are the unadjusted *p*‐values. ‘Corrected *p*‐values’ have been adjusted for sex, birthweight and feeding type. All *p*‐values were calculated using generalized linear models comparing high and low tertiles of visceral fat at 2 years of age. Fold change is calculated relative to the ‘low’ group. Putative annotations: C_24_H_30_O_8_ – Esterone Glucoronide, C_27_H_38_O_9_ –Hydroxyprogesterone Glucoronide, C_30_H_40_O_8_ – Hydroxypentaoxolanostenoic acid.

Abbreviations: LysoPA, lysophosphosphatidic acid; LysoPE, lysophosphatidylethanolamine; LysoPG, lysophosphatidylglycerol; LysoPS, lysophosphatidylserine.

### 3.2 Difference between boys and girls

We found differences in the association between the 15 metabolite variables and the T:P‐ratio between boys and girls (Figure S1). In boys, the model had a predictive performance of 32.2% between individuals in the highest and lowest tertile of T:P‐ratio at 2 years (R^2^X = 0.204, R^2^Y = 0.595, Q^2^ = 0.322, CV‐ANOVA = 3.05 × 10^−8^). In girls, the predictive performance of the model was 11.7% (R^2^X = 0.197, R^2^Y = 0.539, Q^2^ = 0.117, CV‐ANOVA = 0.038). In boys, all metabolic variables except for one unknown metabolite variable showed a significant (*p* < 0.05) association with T:P‐ratio at 2 years. Of these 14 metabolic variables, 13 had a fold change greater than 1, which means that the relative abundance of the metabolic variables were higher in boys with high T:P‐ratio compared to boys with low T:P‐ratio. In girls, 5 out of 15 metabolic variables were significantly associated with T:P‐ratio (Table 3). Four of these had a fold change greater than 1. An unknown metabolite variable had an fold change of 0.79 (*p* = 0.006) in girls, meaning that this metabolite variable had a lower relative abundance in girls with a high T:P‐ratio, compared to girls with a low T:P‐ratio. It was the only metabolic variable that was stronger associated with T:P‐ratio in girls compared to boys.

### 3.3 Effect of feeding type

T:P‐ratio at 2 years of age was not different between infants with exclusive breastfeeding (EBF), exclusive formula feeding (EFF) and mixed feeding. Of the 15 metabolite variables, lysophosphatidylethanolamine (20:1) (LysoPE(20:1)) and the isotope of Lysophosphoglycerol (16:0) (LysoPG(16:0)) were significantly associated with feeding type (Table 5). LysoPE(20:1) had a higher relative abundance in EBF infants and LysoPG(16:0) had an higher relative abundance in EFF infants.

**TABLE 5:** Relative abundance of 15 plasma metabolite variables in different feeding types

	Relative Abundance			Difference
	Breast	Mixed	Formula	*p*‐value
LysoPS(22:2)	1.57	1.57	1.68	0.505
Unknown	0.88	0.94	0.99	0.357
Dimethylarginine	3.92	3.98	4.59	0.338
C_24_H_30_O_8_	6.83	7.31	8.19	0.202
C^13^ isotope of C_30_H_40_O_8_	8.30	7.52	7.62	0.351
C_27_H_38_O_9_	35.97	38.51	38.76	0.442
Unknown	2.83	2.91	3.30	0.159
LysoPE(20:1)	48.04	44.90	43.70	**0.002**
Unknown	27.92	27.69	31.88	0.345
LysoPG(16:0)	6.10	7.23	7.40	**0.001**
C_30_H_40_O_8_	13.20	12.92	14.14	0.684
LysoPA(22:1)	8.84	8.67	9.77	0.452
Unknown	1.88	1.91	1.92	0.568
Unknown	4.09	3.92	3.77	0.702
Unknown	1.07	1.18	1.15	0.193

*Note*: All *p*‐values were calculated using generalized liner models comparing relative abundance in exclusive breast fed, mixed and exclusive formula fed infants. Significant *p*‐values are boldfaced. Relative abundance is shown in arbitrary units. Putative annotations: C_24_H_30_O_8_ – Esterone Glucoronide, C_27_H_38_O_9_ –Hydroxyprogesterone Glucoronide, C_30_H_40_O_8_ – Hydroxypentaoxolanostenoic acid.

Abbreviations: LysoPA, lysophosphosphatidic acid; LysoPE, lysophosphatidylethanolamine; LysoPG, lysophosphatidylglycerol; LysoPS, lysophosphatidylserine.

## 4 DISCUSSION

Our data show that the plasma metabolite profile at 3 months of age can modestly predict body composition at 2 years of age, based on truncal:peripheral fat ratio (T:P‐ratio), with a predictive value of 75.8%, sensitivity of 100% and a specificity of 50%. The predictive value was better in boys than in girls. Of the 15 metabolite variables at 3 months of age that were most strongly associated with the T:P‐ratio at 2 years, 11 were also associated with visceral fat at 2 years of age.

We are the first to describe potential biomarkers at 3 months of age which are associated with body composition outcome at 2 years of age. It has been reported that metabolic biomarkers are different in lean children and children with obesity, with especially branched chain amino acids (BCAAs) concentrations being higher in individuals with obesity.^28^ Second, metabolic biomarkers have been reported to be potentially predictive for unfavourable metabolic outcome in adults, adolescents and school children with overweight and obesity.^29^, ^30^ However, it has never been described before that plasma metabolite profile at 3 months of age are associated with future visceral fat and proxy of body composition, such as skinfold measurements,^4^, ^31^ in a large cohort of healthy infants.

Our results support the hypothesis of a critical window of adiposity programming in early life.^3^, ^4^ It has been reported that growth and body composition, especially in the first 6 months of life, are important for the development of body composition later in life and for the adult metabolic profile.^3^, ^9^ We now add that the plasma metabolic profile in early life is potentially involved in the adiposity programming and contributes to adiposity at 2 years of age. Our results show that only four of the associated plasma metabolites associated with T:P‐ratio at 2 years were also associated with T:P‐ratio at 3 months of life. This means the plasma metabolites we found are independent of the biological progress of body composition.

Of the 15 most strongly associated metabolite variables, 11 were also significantly associated with abdominal visceral fat at 2 years of age, with a fold change below 1. Meaning, infants with high visceral fat at 2 years of age, had lower relative abundance of these metabolite variables at 3 months of age. This is remarkable, since we found the metabolite variables to be associated with T:P‐ratio with a fold change above 1. We found visceral fat and T:P‐ratio not to be correlated at 2 years of age. It has been described that skinfold measurements are correlated with total body fat.^31^ However, this is mostly based on the amount of subcutaneous fat instead of visceral fat.^32^ This could possibly explain, why we found opposite association between T:P‐ratio and visceral fat. Second, it has been described that especially excessive visceral fat is associated with an unfavourable metabolic outcome, with more insulin resistance and a higher risk of diabetes mellitus type II.^12^, ^33^ Since we found an association between metabolite variables at 3 months of age with visceral fat at 2 years, our findings could suggest that metabolite profile in early life is also important for programming the metabolic outcome later in life.

Our results showed that plasma dimethylarginine and lysophosphatidylserine 22:2 (LysoPS 22:2) at 3 months of age are highly correlated with body composition outcomes at 2 years. In adults, higher dimethylarginine levels have been associated with dyslipidemia and accelerated atherosclerosis and found to be predictive for cardiovascular events.^34^, ^35^ One research group described dimethylarginine levels to be higher in teenagers obesity compared to their lean peers.^36^ LysoPS 22:2 has been mainly studied in rodent and in vitro studies. These studies show that LysoPS is involved in glucose uptake in muscle and adipose tissue.^37^, ^38^ In contrast, it has been reported that LysoPS levels are lower in hepatic tissue from adults with obesity, compared to lean peers.^39^ Since we found LysoPS to be associated with visceral fat at 2 years of age with a fold change below 1, the mechanism for LysoPS involved with intra‐abdominal fat could possibly differ from subcutaneous fat. We also found plasma lysophosphatidic acid(22:1) (LysoPA (22:1)) at 3 months of age to be associated with body composition outcome at 2 years. LysoPA has been described to interact with mTOR signalling, affecting body composition due to changes in lean body mass.^40^

Dimethylarginine, LysoPS and LysoPA have been associated with inflammatory processes.^41^, ^42^, ^43^ It was reported that LysoPS is an emerging class of signalling compounds^44^ and could be interacting with Toll‐like receptor dimers (TLR2/6).^45^ LysoPA has been reported to activate peroxisome proliferator‐activated receptor gamma (PPAR‐γ).^46^ Both Toll‐like receptors and PPAR‐γ are known to be involved in the development of inflammation, obesity and metabolic syndrome.^47^, ^48^ Second, LysoPS, LysoPA, lysophosphatidylethanolamine (LysoPE) and lysophosphatidylglycerol (LysoPG) are all deacylated products of phospholipids and are the result of phospholipase A activity.^46^ Phospholipase A2 is important in lipid metabolism and it has been described that serum levels of phospholipase A2 are increased in patients with obesity and inflammation, due to activation of pro‐inflammatory pathways in preadipocytes.^49^, ^50^ Our findings could, therefore, suggest that the identified metabolites are involved in adiposity development and systematic low grade inflammatory processes from early age onwards.

Plasma metabolite variables at 3 months of age had a higher predictive value in boys than in girls. We found no differences between girls and boys in skinfold measurements and abdominal fat distribution at 2 years of age. However, it has been described that girls have a higher fat mass percentage and lower lean body mass, measured with air displacement plethysmography compared to boys.^7^ One research group also found differences in metabolic biomarkers associated with insulin resistance between female and male adolescents with obesity.^51^ Our findings of metabolite differences already present at a very young age suggest that these metabolite variables may have different mechanism of action in the adiposity programming of boys and girls.

Two of the 15 metabolite variables showed a different relative abundance in infants with different feeding types. This is in line with our previous findings in other cohorts, where we identified differences in lipid profile between infants receiving exclusive breastfeeding compared to infants receiving formula feeding.^18^, ^20^, ^52^ Since only 2 of the 15 metabolite variables were different across different infant feeding types, the associations we found between metabolite variables at 3 months of age and body composition outcome at 2 years of age seemed independent of infant feeding type.

This is the first study reporting plasma metabolite profile at 3 months that are associated with body composition outcome at 2 years. The strengths of our study are the longitudinal body composition measurements and collected blood samples in a large group of healthy infants combined with a validated technique to obtain a detailed metabolic profile. We acknowledge some limitations. Although we did not annotated all identified metabolite variables, with the level of detail we provide, others can replicate our work and future annotation is possible. We identified metabolic variables in a single cohort containing a very low number of infants developing overweight and no infants developed obesity at the 2 years of age. It was, therefore, not possible to predict obesity based on metabolic profile. The metabolite variables we found, were associated with childhood body composition trajectories. They will, therefore, have to be validated in infants who develop overweight or obesity and in an independent external cohort to validate generalizability in healthy infants.

In conclusion, we found that the plasma metabolite profile at 3 months of age can modestly predict body composition at 2 years of age, measured as T:P‐ratio. The predictive value was higher in boys than in girls. Of the 15 highest correlated plasma metabolite variables, 11 were also associated with visceral fat at 2 years. These findings contribute to our insight into the adiposity programming in the first months of life.

## CONFLICT OF INTEREST

BJMvdH is an employee of Danone Nutricia Research.

## AUTHOR CONTRIBUTIONS

Albert Koulman, Anita Hokken‐Koelega, Susanne Brix, Ken Ong and David Dunger were in charge of designing the study. Inge van Beijsterveldt, Kirsten de Fluiter and Anita Hokken‐Koelega were in charge of the cohort, design and collecting of the data and samples. Stuart Snowden and Albert Koulman performed the metabolomics and bioinformatic analysis. Drafting the manuscript was primarily done by Inge van Beijsterveldt and Stuart Snowden under supervision of Anita Hokken‐Koelega and Albert Koulman. All authors were involved in writing the manuscript and had final approval of the submitted version.

## Supporting information


**Figure S1.** Partial least squares – discriminant analysis modelling of the relationship between metabolite profile and high and low truncal: peripheral fat ratio in males and females separately.
*Female (R* ^*2*^ *X = 0.197, R* ^*2*^ *Y = 0.539, Q* ^*2*^ *= 0.117, CV‐ANOVA = 0.038) Male (R* ^*2*^ *X = 0.204, R* ^*2*^ *Y = 0.595, Q* ^*2*^ *= 0.322, CV‐ANOVA = 3.05* × *10* ^*−8*^ *)*
**Table S1.** Panel of 15 plasma metabolite variables at 3 months associated with truncal: peripheral fat ratio at 3 months
