# Supplementary material for: Metabolomics in early life and the association with body composition at age 2 years
Source: Pediatr Obes. 2021 Oct 13;17(3):e12859. doi: 10.1111/ijpo.12859 (PMC9286420; doi:10.1111/ijpo.12859)
Supplement: Supplementary file 1 — Figure S1. Partial least squares – discriminant analysis modelling of the relationship between metabolite profile and high and low truncal: peripheral fat ratio in males and females separately. Female (R 2 X = 0.197, R 2 Y = 0.539, Q 2 = 0.117, CV‐ANOVA = 0.038) Male (R 2 X = 0.204, R 2 Y = 0.595, Q 2 = 0.322, CV‐ANOVA = 3.05 × 10 −8 ) Table S1. Panel of 15 plasma metabolite variables at 3 months associated with truncal: peripheral fat ratio at 3 months [file IJPO-17-0-s001.pdf]

Metabolomics in early life and the association with body composition at age 2 years - Inge van Beijsterveldt, Stuart Snowden, Pernille Neve Myers, Kirsten de Fluiter, Bert van de Heijning, Susanne Brix, Ken Ong, David Dunger, Anita Hokken-Koelega, Albert Koulman - University of Cambridge, UK
Tel: +44 (0) 1223 763202, e-mail: ak675@medschl.cam.ac.uk / a.hokken@erasmusmc.nl

Supplement Figure 1. Partial least squares – discriminant analysis modeling of the relationship between metabolite profile and high and low truncal:peripheral fat ratio in males and females separately.

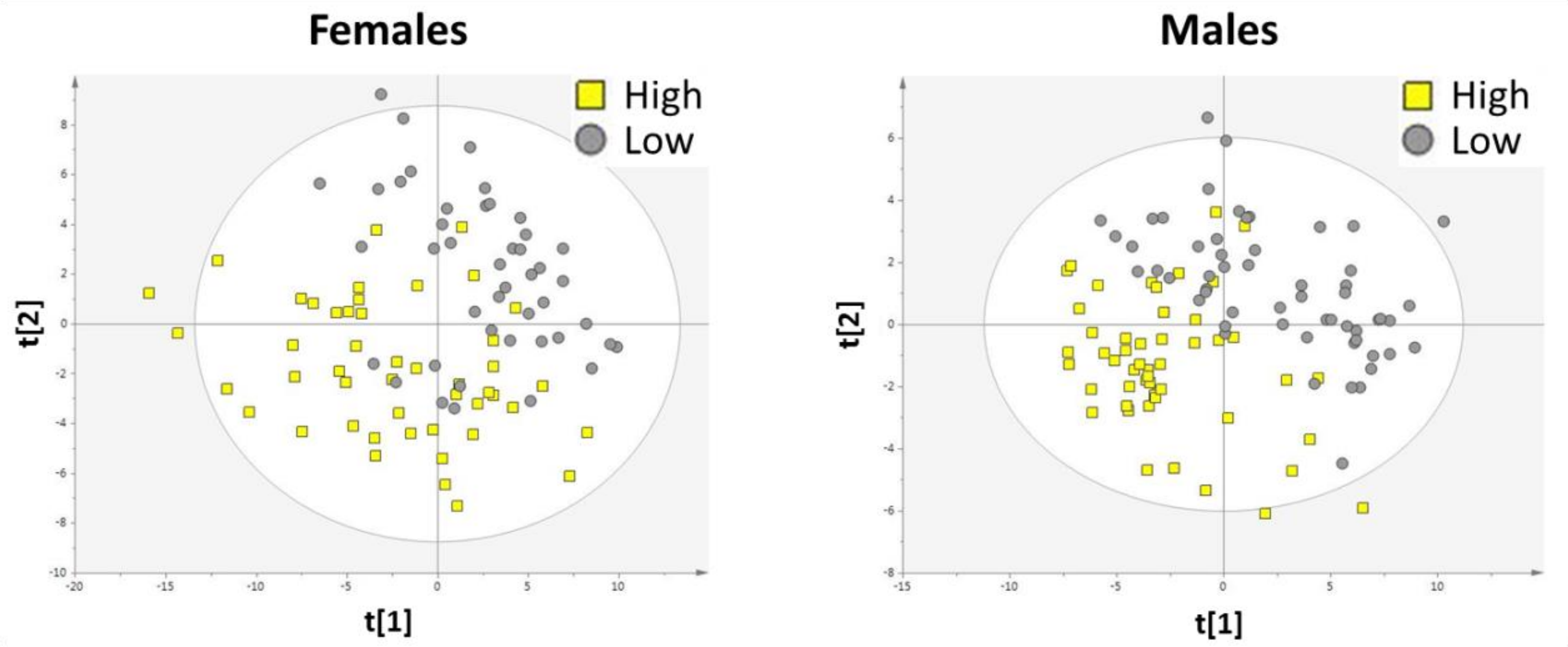

Female (R^2X = 0.197, R^2Y = 0.539, Q^2 = 0.117, CV-ANOVA = 0.038) Male (R^2X = 0.204, R^2Y = 0.595, Q^2 = 0.322, CV-ANOVA = $3.05x10^{-8}$)

Metabolomics in early life and the association with body composition at age 2 years - Inge van Beijsterveldt, Stuart Snowden, Pernille Neve Myers, Kirsten de Fluiter, Bert van de Heijning, Susanne Brix, Ken Ong, David Dunger, Anita Hokken-Koelega, Albert Koulman - University of Cambridge, UK
Tel: +44 (0) 1223 763202, e-mail: ak675@medschl.cam.ac.uk / a.hokken@erasmusmc.nl

Supplement Table 1. Panel of 15 plasma metabolite variables at 3 months associated with truncal:peripheral fat ratio at 3 months

	All Individuals			Boys		Girls	
**Metabolite variables**	**p-value**	**Corrected p-value**	**Fold change**	**p-value**	**Fold change**	**p-value**	**Fold change**
**LysoPS(22:2)**	0.150	0.107	1.15	0.241	1.17	0.353	1.13
**Unknown**	0.074	0.058	1.25	0.114	1.31	0.310	1.21
**Dimethylarginine**	0.042	0.029	1.39	0.124	1.42	0.171	1.38
**$C_{24}H_{30}O_8$**	0.070	0.051	1.28	0.074	1.41	0.361	1.20
**C^{13} isotope of $C_{30}H_{40}O_8$**	0.026	0.014	1.20	0.009	1.33	0.542	1.07
**$C_{27}H_{38}O_9$**	0.051	0.038	1.18	0.118	1.20	0.180	1.18
**Unknown**	0.454	0.330	1.07	0.292	1.15	0.811	1.03
**LysoPE(20:1)**	0.147	0.061	1.04	0.269	1.04	0.279	1.05
**Unknown**	0.067	0.049	1.27	0.050	1.41	0.399	1.18
**LysoPG(16:0)**	0.034	0.041	1.12	0.041	1.15	0.270	1.10
**$C_{30}H_{40}O_8$**	0.013	0.009	1.53	0.013	1.80	0.237	1.34
**LysoPA(22:1)**	0.167	0.126	1.19	0.186	1.23	0.447	1.16
**Unknown**	0.465	0.318	0.98	0.390	0.97	0.643	0.98
**Unknown**	0.088	0.078	1.47	0.095	1.66	0.498	1.25
**Unknown**	0.097	0.164	1.08	0.272	1.06	0.327	1.08

'p-value' are the unadjusted p-values. 'Corrected p-values' have been adjusted for sex, birthweight and feeding type. All p-values were calculated using generalized linear models comparing high and low tertiles of trunk:peripheral ratio at 3 months of age. Fold change is calculated relative to the 'low' group. Putative annotations: $C_{24}H_{30}O_8$ – Esterone Glucoronide, $C_{27}H_{38}O_9$ –Hydroxyprogesterone Glucoronide, $C_{30}H_{40}O_8$ – Hydroxypentaoxolanostenoic acid. Abbreviations: LysoPA= lysophosphatidic acid, LysoPE= ysophosphatidylethanolamine, LysoPG=lysophosphatidylglycerol, LysoPS= lysophosphatidylserine

Metabolomics in early life and the association with body composition at age 2 years - Inge van Beijsterveldt, Stuart Snowden, Pernille Neve Myers, Kirsten de Fluiter, Bert van de Heijning, Susanne Brix, Ken Ong, David Dunger, Anita Hokken-Koelega, Albert Koulman - University of Cambridge, UK
Tel: +44 (0) 1223 763202, e-mail: ak675@medschl.cam.ac.uk / a.hokken@erasmusmc.nl
